# Optimizing Dog Rabies Vaccination Services to the Public: A Discrete Choice Experiment in Guangdong, China

**DOI:** 10.3390/ani13111767

**Published:** 2023-05-26

**Authors:** Ruiqi Chen, Yingxin Zeng, Zhile Deng, Hongfu Liu, Manyi Chen, Yaoming Liang

**Affiliations:** 1 College of Economics & Management, South China Agricultural University, No. 483 Wushan Road, Tianhe District, Guangzhou 510642, China; rickychen@stu.scau.edu.cn (R.C.); yxzeng02042009@163.com (Y.Z.); lhf1192896646@163.com (H.L.); 2 College of Veterinary Medicine, South China Agricultural University, Guangzhou 510642, China; scauzhile@163.com; 3 Nanling Corridor Country Revitalization Institute, Xiangnan University, Chenzhou 423000, China

**Keywords:** rabies, vaccination, preference, discrete choice experiment

## Abstract

**Simple Summary:**

A discrete choice experiment was designed to explore dog-keeping households’ preferences for dog rabies vaccination services. Dog-keeping households can be classified into three types based on the latent class model: resolute executors, mischievous rebels, and incentivized compliers. The residence, children in the household, perception of the safety risks, and knowledge of rabies may contribute to the heterogeneity among the households. Supportive measures should be provided to improve the convenience of dog rabies vaccination services in emerging countries like China.

**Abstract:**

Vaccination for dogs is essential for controlling rabies and achieving the goal of eliminating dog-mediated rabies globally by 2030. This paper aims to investigate the preferences for public services regarding rabies vaccination, in an effort to optimize the existing rabies vaccination and prevention programs in China. The households investigated had significant preferences for dog rabies vaccination service attributes. The households can be classified into three types: resolute executors (52.13%), mischievous rebels (5.85%), and incentivized compliers (42.02%). The residence, the presence of children in the household, perception of the safety risks, and knowledge of rabies may be sources of heterogeneity. Supportive services on dog rabies vaccination should be made available, such as arranging weekend vaccination services, building mobile vaccination stations, providing home vaccination services, and increasing vaccine supply through multiple channels. Furthermore, multiple measures can be taken to increase rabies vaccination awareness among family members and facilitate dog management innovation to further increase the level of rabies prevention and control.

## 1. Introduction

Rabies is a fatal zoonotic disease caused by the rabies virus that invades the central nervous system. The disease has an almost 100% case fatality rate, and it threatens more than 150 countries and regions worldwide, with Asia having the highest number of cases, followed by Africa. Despite the invention of the rabies vaccine by Louis Pasteur as early as 1886, the World Health Organization (WHO) estimates that 59,000 people still die of rabies annually (source: Centers for Disease Control and Prevention, National Center for Emerging and Zoonotic Infectious Diseases, Division of High-Consequence Pathogens and Pathology), with children accounting for about 40% of the cases [1,2,3,4]. Given that the prevention and control of rabies is crucial for human well-being, the 28 September was officially designated as World Rabies Day in 2007 by the WHO, the World Organization for Animal Health (WOAH), and the United States Centers for Disease Control and Prevention. In December 2015, these organizations, together with the Food and Agriculture Organization of the United Nations, and the Global Alliance for Rabies Control, held the International Conference on the Elimination of Rabies in Geneva. The conference set ambitious goals to eliminate dog-mediated human rabies and achieve zero case worldwide by 2030 [5].

**Figure 1:**
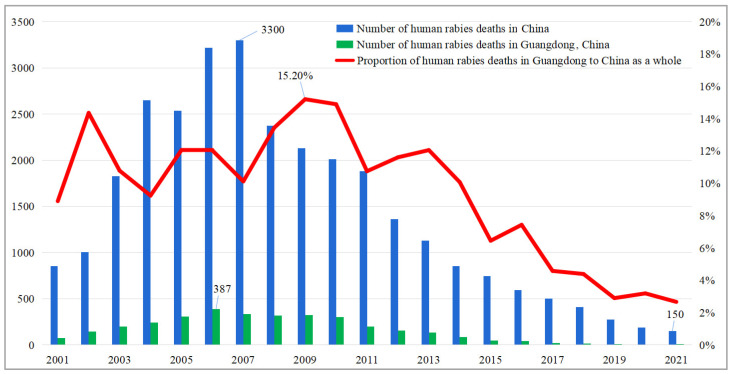
Trends in human rabies deaths from 2001 to 2021. (Source: China Center for Disease Control and Prevention, Guangdong Provincial Health Commission. Mortality = number of deaths in the year/number of cases in the year. The national data do not include data from Hong Kong, Macao, or Taiwan).

China is among the countries most affected by human rabies, with deaths related to the disease being reported in all 31 of its provincial administrative regions as of 2021. In response, in 1980, four statutory bodies, namely the Ministry of Health, the Ministry of Agriculture and Forestry, the Ministry of Foreign Trade, and the National Supply and Marketing Cooperation, jointly issued the “Notice on the Control and Elimination of Rabies” and “Dog Management Regulations”. Following the decree on the “Animal Epidemic Prevention Law” in 1998, detailed implementation rules were established throughout the country, with major cities adopting standardized measures for dog management, including compulsory rabies vaccination and pet registration. As a result of these efforts, and the increase in rabies immunity nationwide, the incidence of rabies in China has been declining annually since 2007, when the country reached the highest incidence of the disease this century (with 3300 cases per year). The total number of rabies-related deaths in the country dropped to 150 in 2021, as shown in Figure 1.

Despite China’s significant progress in the prevention and control of rabies in recent years, the situation remains challenging. On one hand, rabies cases are widely dispersed across the country, often occurring in rural areas, making centralized prevention and control management difficult. On the other hand, the general public still lacks awareness of the risks and prevention measures for rabies. A survey conducted by Yang et al. [6] of 1906 students in three rural middle schools in Guangxi found that only 12.01% of the students recognized their vulnerability to rabies, 21.51% knew they should be vaccinated immediately after being bitten by dogs, and 13.69% were aware of preventive measures against rabies. In a study of 1015 patients, who had been bitten by animals, in a rabies prevention clinic in Wuhan, Li et al. [7] found that only 56.85% of respondents knew that rabies is infectious. More than 20% of respondents believed that rabies vaccination for dogs and cats was unnecessary, and about 70% of participants reported that they never needed reminders to get vaccinated after being bitten.

Currently, research on rabies and its prevention and control in China is primarily focused on natural sciences, such as virus infectious mechanisms, vaccine development, and epidemiology, with limited studies in the fields of humanities and social sciences. According to Miao et al. [8], post-exposure prophylaxis is not only expensive but also ineffective in preventing the spread of rabies from dogs to humans and other susceptible animal species compared to large-scale dog vaccinations. However, rabies control in China has become polarized, resulting in excessive vaccinations for registered dogs, but a lack of regulation for unregistered dogs [9]. Although dog registration and rabies vaccination are mandatory, neither has been strictly implemented, and accurate statistical data on registration are lacking [10]. Furthermore, while vaccination coverage in some major cities in China exceeds the recommended rate of 70% by the WHO, it is still insufficient to eliminate rabies epidemics, as unregistered or stray dogs and other rabies hosts are easily neglected [11,12]. Therefore, regulating dog registration and increasing the immunization rate for dogs remain some of the most effective measures to achieve the goal of eliminating human rabies in China.

To achieve this, it is crucial to provide rabies vaccination programs that are well received by the public. Identifying and responding to public demand for rabies vaccination for dogs is essential. However, limited empirical studies have investigated this topic. This study surveyed 633 dog-keeping households across 21 cities in Guangdong province, which is located in southern China, between latitude 20°09′~25°31′ N and longitude 109°45′~117°20′ E, covering an area of 179,700 square kilometers. The study had three main objectives: to explore the preferences of dog-keeping households on the attributes of rabies vaccination, to analyze the heterogeneity of the dog-keeping households’ preferences for vaccination services using a mixed logit model and the latent class model, and to propose suggestions for optimizing local rabies vaccination-related services in emerging countries like China. The findings provide an important reference for improving the rabies immunization rate for dogs and contributing to the achievement of the 2030 goal.

## 2. Methods and Materials

### 2.1. Discrete Choice Experiment Design

The discrete choice experiment (DCE) is a frequently used method for optimizing medical intervention programs [13]. This approach is widely utilized to investigate public preferences for the attributes of a particular product or service, as demonstrated by studies such as McPhedran et al. [14] and Makabayi-Mugabe et al. [15]. In the DCE, respondents are presented with a series of choices between two or more options that differ in various dimensions, or “attributes”, each of which has multiple “levels”. By analyzing the choices made by participants, researchers can infer the utility value of different attributes and levels for different groups [16,17], allowing them to understand the relative importance and impact of policies on different sectors of society. This, in turn, can help predict the degree of public support for specific policies [18]. Additionally, evaluating respondents’ preferences for policies may also involve measuring their willingness to pay (WTP), which can reveal the range of public preferences in favor of certain products or services.

This paper utilized the DCE methodology to examine the preferences of dog-keeping households for dog rabies vaccination services. We selected attributes for the experiment based on common factors associated with the use of the rabies vaccine for dogs, such as time, place, appointment, origin of vaccine, subsidy, and price (see Table 1). Specifically, attributes such as time, place, and appointment reflect the accessibility of the vaccination services. The origin of the vaccine, either domestically produced or imported, reflects the respondents’ trust in the safety and quality of the vaccine. The subsidy level reflects the degree of concern shared by local authorities and society about rabies prevention and control. Many domestic organizations and departments frequently engage in “public service” activities to provide free or discounted rabies vaccines for dogs for the general public. We set the subsidy level at 25%, 50%, and 75% to make it easier for respondents to calculate the discounts in the DCE and distinguish the subsidy levels to a greater extent. The prices for dog rabies vaccination services are primarily based on the average market price in rural areas (25 CNY/needle), and we set them at four levels, namely 25, 50, 75, and 100 CNY/needle, in an arithmetical manner.

Accordingly, 2 × 3 × 2 × 2 × 3 × 4 = 288 possible product or service options could be obtained, and 288 × 287/2 = 41,328 combinations or choice sets could be generated. However, when there are three or more factors involved, interactions between the factors may increase the complexity of the experiment, making it difficult to implement. Therefore, we utilized the Ngene version 1.2.1 software package to design 36 choice sets based on the D-optimal fractional factorial experiment design method to estimate the utility of the attributes of dog rabies vaccination services for dog-keeping households. These choice sets were divided into 6 groups with 6 choice tasks in each group to minimize the probability of choice fatigue.

**Figure 2:**
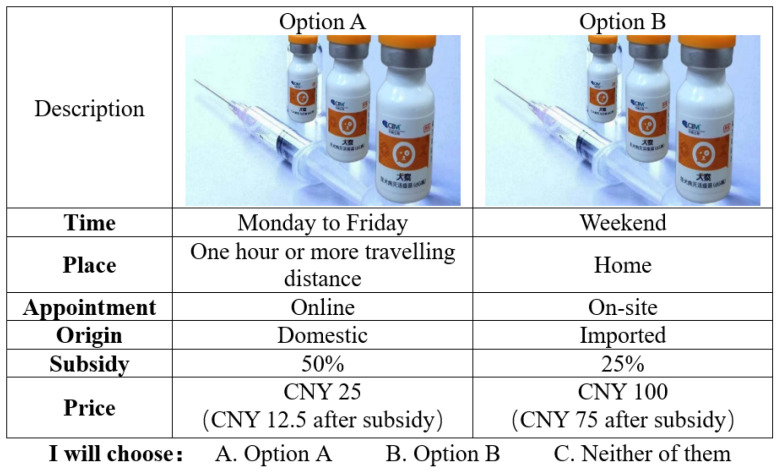
A sample of the choice sets.

In the DCE, each choice set comprised of two hypothetical alternatives and an opt-out or “no vaccination” option to make the experiment more realistic. Respondents were assigned to different choice scenarios randomly based on their birth months. For example, respondents born in January or July received the first group of choice tasks, while those born in February or August received the second group, and so on. A cheap talk script was provided before the experiment to reduce the hypothetical bias of the respondents [19] and ensure that the data quality was acceptable. Figure 2 displays one of the choice sets.

### 2.2. Survey Design

Guangdong province was selected as the research site for our study due to several reasons. Firstly, Guangdong has a high incidence of rabies historically, with 3365 deaths between 2001 and 2021, accounting for 11.22% of the country, and reaching a peak period in 2006 with 387 cases of illness and death, equivalent to 11.80% of the country that year (as shown in Figure 1). Secondly, Guangdong has the largest population of pet-keeping households in China, accounting for 10.87% of the country in 2017 (source: Beijing LinkApp Technology Co., Ltd., Beijing, China, (http://linkip.cn (accessed on 30 March 2023))), with the number of pet dogs expected to reach 5.9 million in 2021. (The number of dogs and cats in China exceeds 112 million, including 54.29 million pet dogs, according to the 2021 China Pet Industry White Paper). Thirdly, Guangdong has a large number of pet-related enterprises, with 55,273 new pet-related enterprises registered in 2021, accounting for 5.76% nationwide, and 234,162 by 15 August 2022, accounting for 9.41% of the country. (We used “pet” as the keyword and searched in the National Enterprise Credit Inquiry System (https://www.qcc.com/ (accessed on 30 March 2023)) for pet-related enterprises in China and around the world. The results show that as of 15 August 2022, the number of pet-related enterprises in Guangdong is second only to Fujian (288,558) and Jiangxi (286,002)). Finally, the diverse economic classes in Guangdong contribute to a stronger willingness for pet-related consumption, with an average consumption in pet-keeping households of CNY 600–1000 per month, higher than the national average of CNY 200–500 (retrieved from https://www.sohu.com/a/231803695_100086638 (accessed on 31 October 2022)). Furthermore, Guangdong covers 2 first-tier cities, 5 second-tier cities, and 14 cities below the third tier, and its GDP has ranked first for 33 consecutive years in China up to 2021. Therefore, our study provides valuable insights into the preferences of Chinese dog-raising households for dog rabies vaccination services through a survey of households in Guangdong province.

We utilized a hybrid field- and web-based research method for the survey due to the strict quarantine isolation regulations enforced in China during the COVID-19 outbreak. In addition, face-to-face surveys could significantly increase the survey costs and lead to bias caused by respondents’ limited cognitive resources, including time and energy. The survey was conducted anonymously with ethical approval from the College of Veterinary Medicine at South China Agricultural University. The respondents were households that either kept dogs or had dog-petting experiences within the past two years. The aim of the survey was to investigate the basic information from households, their dog-keeping experiences and risk perception, as well as their knowledge on rabies prevention and control.

In August 2022, a survey team was formed to conduct a pre-test in the cities of Yangjiang and Wuchuan, as part of our research project. During the pre-test, it was noted that some respondents had difficulty understanding certain terminology related to rabies and other specialized terms used in the questionnaire. As a result, we made several adjustments to the questionnaire. Firstly, we rephrased the questionnaire terms to make them more concise and easier to understand. Secondly, we eliminated the survey questions that were not relevant to the local situation and added some more valuable questions. Finally, we adjusted the structure of the questionnaire to allow respondents to answer the questions more fluently. These changes were made to ensure that the survey instrument was clear and effective in collecting the necessary data for our study.

**Figure 3:**
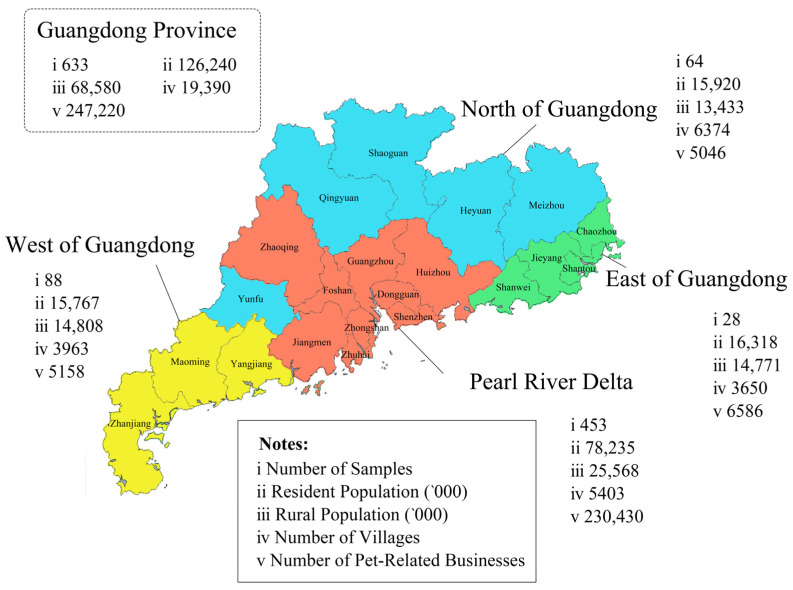
Survey locations. (Source: the Guangdong Statistical Yearbook 2021, the Guangdong Rural Statistical Yearbook 2021, the official government websites of various regions in Guangdong province, the Yigecun platform (http://www.yigecun.com/ (accessed on 30 March 2023)), the National Enterprise Credit Inquiry System (https://www.qcc.com/ (accessed on 30 March 2023))).

After conducting the pre-test, we proceeded with a formal survey in 21 cities within the province using both online and offline formats (refer to Figure 3). Each respondent who participated in the field survey was provided with a daily necessity award worth approximately CNY 5 (equivalent to about USD 0.731, based on the exchange rate of the dollar against the RMB (1: 6.8361) on 31 December 2022). For the online survey, we utilized the services of Wenjuanxing, a professional online survey platform in China. This platform maintains a group of consumers who participate in surveys periodically for small incentives. The participants were invited to the survey through email invitations and URLs, and received rewards in the form of credits which could be converted into vouchers for shopping. Participation in the survey was voluntary. Wenjuanxing’s sample service includes a rigorous quality control mechanism, such as sample quality control, filler control, filling process control, and whole tracking effect, to ensure that the recovered response data are true and valid.

To achieve statistical significance and to meet the DCE rank condition, we followed the rules commonly used in choice experimental designs [20,21] to determine the minimum sample size:

$$N\geq 500\times \left(\frac{L}{A\times C}\right)=500\times \left(\frac{4}{2\times 6}\right)=166.667$$

Specifically, N is the total samplings, L the number of strata with the highest level of hierarchy in the study attributes, A is the number of choice options in a choice set, and C is the number of choice sets faced by each respondent. Given that we divided the 36 choice sets into 6 groups for the study, the minimum sample size for the DCE was calculated to be 167. In August 2022, we received a total of 679 completed questionnaires. We used completeness and quality of information as the screening criteria and excluded invalid questionnaires with key information missing or logical basis. Respondents with a single response behavior were also excluded, as they may not have read the questions completely and may only have completed the survey for the reward. Ultimately, we obtained 633 valid questionnaires, with an effective rate of 93.23%, and 3798 completed choices (633 respondents × 6 choices), far exceeding the minimum sample size requirement.

### 2.3. Sample Description

#### 2.3.1. Sample Characteristics

All the statistical analyses in this paper were conducted using the Stata 17.0 software. Table 2 displays the sampling characteristics of the survey. Of the respondents, 63.51% were female with an average age of 29.156 years. Additionally, 66.51% of the respondents held a university degree or higher, and 14.53% worked in animal-related jobs. As there has been no census on domestic dogs for a significant period of time, the statistics were mainly collected from the pet industry. The results indicated that rural dog-keeping households accounted for 29.54%, while urban dog-keeping households accounted for 70.46%. The annual household income of the surveyed dog-keeping households was evenly distributed, with 55.45% reporting less than CNY 100,000 (equivalent to about USD 14,620, based on the exchange rate of the dollar against the RMB (1: 6.8361) on 31 December 2022). Of the respondents, 13.59% reported living alone, and 45.81% reported having children under 12 in their dog-keeping households.

#### 2.3.2. Dog Keeping Conditions and Management

Table 3 provides an overview of the dog-keeping and management practices in the surveyed households. Among the respondent households, 77.57% kept one domestic dog, 15.96% kept two, and 6.48% kept three or more dogs. The majority (68.72%) have owned their pets for less than four years, while 31.28% have owned them for more than five years. The source of the dogs can be traced back to purchases from markets, pet stores, or other dog-keeping households (50.87%), while some dogs were received as gifts from friends or relatives (36.65%). Precautionary information about the risks from dog-keeping was received by 76.15% of the dog-keeping households, either from the buyers or givers. Regarding the reasons for keeping a dog, 71.72% of the households kept them for family companionship, 63.03% for personal preference, and 39.18% for home safeguarding.

In terms of the management of domestic dogs, 38.39% of the surveyed households did not impose social and spatial restrictions, which could increase the risk of dog attacks. However, 79.94% of households complied with the annual dog vaccination requirements. The remaining 20.06%, who did not adhere to the regular dog vaccinations, were the focus of rabies prevention and control efforts. The top four factors that affect vaccination rates were excessive workload and lack of time for vaccination (42.81%), vaccination sites being too far from home (32.86%), cumbersome procedures (29.38%), and high vaccination costs (27.80%). These four factors were reflected in the attributes of our DCE. About 32.70% of households believed that people should apply to local communities or village committees before keeping a dog, and 46.92% believed that they should register with the local community or village committee. Moreover, 74.09% of households regarded the frequency of rabies prevention and control publicity by local community or village committees as average, less, or inadequate.

#### 2.3.3. Perception of Safety Risks Related to Dogs

Table 4 presents the perception of the safety risks related to dog-keeping households. These risks were measured in terms of life safety and property safety, with five and two items, respectively. The life safety risks included concerns such as “Dog shedding easily causes human allergies”, “People who raise dogs are susceptible to diseases”, “People who eat dog meat are prone to diseases”, “I am worried about being bitten or scratched by a dog”, and “I am worried about my dog biting or scratching others”. The property safety risks included worries about “my dog damaging household items” and “the increased expenses caused by my dog’s illness”. Each item was measured using a 5-point Likert scale ranging from “1” for “completely disagree” to “5” for “completely agree”. Cronbach’s α value for each item related to dog safety risk perception was above 0.6, with a total α value of 0.72, indicating an acceptable reliability coefficient. The safety risk perception variable was obtained through the sum of the scores of the seven items, and we categorized the perception of safety risks as high or low based on the mean value.

#### 2.3.4. Knowledge of Rabies among Households with Dogs

We assessed the extent of the rabies knowledge among households that kept dogs, by examining their understanding of rabies and any potential misconceptions surrounding it. Table 5 displays the descriptive statistics for the responses provided by dog-keeping households to questions related to rabies knowledge. The results indicate that the average percentage of correct answers for the four areas assessed, namely conceptual understanding, hosts and transmission routes, prevention, and treatment, were 81.58%, 86.94%, 89.62%, and 87.42%, respectively. It can be concluded that the level of rabies knowledge among dog-keeping households is relatively high.

Rabies is a zoonotic disease caused by the rabies virus that can be fatal once it invades the central nervous system, with a near 100% fatality rate. However, the severity of the disease was not fully recognized by 27.80% of dog-keeping households. Furthermore, 18.48% of households were unaware that washing the wound with water or soapy water immediately after being bitten is crucial, and 18.01% of households erroneously believed that only dogs can transmit the rabies virus. This suggests that dog-keeping households’ knowledge of rabies needs to be improved, particularly regarding the conceptual understanding, hosts and transmission routes, and treatment.

To evaluate the level of rabies knowledge among dog-keeping households, we assigned values based on their responses to the questions related to conceptual understanding, hosts and transmission routes, and treatment. A value of 0 was assigned if the respondents answered, “don’t know”, while a value of 1 was assigned if their answer was deemed correct. The scores for all the items were added together, and the level of rabies knowledge among dog-keeping households was determined accordingly. We categorized the level of knowledge as either high or low based on the mean score.

## 3. Results and Analyses

### 3.1. Preferences for Dog Rabies Vaccination Services

According to Table 6, the results of the mixed logit model are consistent with those of the conditional logit model. Overall, the respondents had significant preferences for the three attributes for vaccination: time, location, and online appointment. Among them, home vaccination had the highest utility value (β = 1.327). The respondents preferred imported vaccines (β = 0.131) to domestic vaccines. Increasing the subsidy from 25% to 50% motivated the respondents to vaccinate their dogs at a higher rate (β = 0.415), and the incentive can be doubled when the subsidy is increased to 75% (β = 0.855). This indicates that government subsidies can increase public utility scores and promote dog rabies vaccination.

The results also reveal heterogeneity in respondent preferences for dog rabies vaccination services. The standard deviation coefficients for the attributes are statistically significant at least at the 5% level, except for the 50% subsidy. These include weekend vaccination, half-hour traveling distance, home vaccination, online appointment, imported vaccines, and 75% subsidy. Specifically, the preference for home vaccination varied the most, followed by weekend vaccination, imported vaccines, online appointment, 75% subsidy, and half-hour traveling distance.

### 3.2. Heterogeneity Analysis of Dog Rabies Vaccination Service Preferences

The study utilized the latent class model to investigate the heterogeneity of the preferences among households that kept dogs. The first step involved determining the appropriate number of classes, which was achieved by comparing the Bayesian information criterion (BIC) and the consistent Akaike’s information criterion (CAIC). Despite attempts to test four or more classes, a singular covariance matrix rendered these efforts unsuccessful. Based on the information criteria presented in Table 7, a 3-class model was deemed most appropriate for the analysis, as it provided a balance between parsimony and interpretability [22].

Table 8 presents the results of the latent class model. The first section displays the utility coefficients for the dog rabies vaccination service attributes, while the second section lists the classification membership coefficients.

In the first latent class (Class 1), the preferences of households with dogs for most rabies vaccination service attributes were not significant, except for the weekend vaccination and subsidy attributes (which also have the smallest coefficients among the groups). However, the coefficient for the “No vaccination” variable was the largest and significantly negative compared to the other groups. This indicates that this type of household is the most determined to follow the vaccination schedule and does not require much motivation. Therefore, we refer to this type of household as “resolute executors”. This type of household accounts for more than half of the sample (52.13%). The level of knowledge about rabies has a significantly negative impact on the probability of belonging to this type of household.

In the second latent class (Class 2), the preferences of dog-keeping households for most vaccination service attributes were not significant, except for the weekend vaccination attribute, which had the largest and significantly positive coefficient among the groups. However, the coefficient for “No vaccination” was significantly positive, indicating that this type of dog-keeping household may be the least willing to vaccinate their dogs. Fortunately, this type of dog-keeping households accounts for only 5.85% of the sample. Factors such as having children in the household, a low perception of the safety risks, and a low knowledge about rabies are likely to increase the probability of belonging to Class 2. Therefore, we refer to this type of household as “mischievous rebels”.

In the third latent class (Class 3), most of the utility coefficients for attributes were significant at the 1% level and had relatively high values. This group of dog-keeping household is highly sensitive to price and other non-price attributes. They are more likely to choose a vaccination plan when certain incentives are provided. Therefore, we define this type of dog-keeping household as “incentivized compliers”, accounting for 42.02% of the sample. This group may have a relatively high perception of the safety risks and knowledge about rabies.

Table 9 provides a more detailed comparison of the social characteristics for the three identified classes. The proportion of households with a high perception of the safety risks is highest in Class 1 (60.91%), compared to Class 2 (29.73%) and Class 3 (51.50%). In terms of households with children under 12 years old, Class 2 has the highest proportion (75.68%), which is significantly higher than Class 1 (49.70%) and Class 3 (56.77%). Class 3 has the highest proportion of urban residents (75.19%), and the proportion of households with a high knowledge level is significantly higher (77.82%) compared to Class 1 (52.73%) and Class 2 (45.95%).

To further evaluate the robustness of the latent class model estimation results, the model was re-estimated in three dimensions. Specifically, variables related to having children in the household, safety risk perception, and knowledge of rabies were removed in models 1, 2, and 3, respectively. The estimation results are presented in Table 10.

Upon comparing the estimation results presented in Table 8 with those in Table 10, it can be observed that the utility function coefficients have experienced slight changes after removing the variables related to having children in the household, safety risk perception, and knowledge of rabies. However, these changes are not significant and the overall trends remain consistent. This finding suggests that dog-keeping households’ preferences for dog rabies vaccination services are highly stable. Additionally, the classification membership variables were found to have limited impact on utility, despite their ability to explain some of the heterogeneity in the respondents’ preferences. This further confirms that the utility derived by dog-keeping households during the DCE is primarily influenced by their selection of dog rabies vaccination service attributes [23].

The estimated results for the membership function coefficients are consistent with those shown in Table 8. When the variable “having children in the household” was removed from Model 1, the signs and significance of the other variables remained consistent with the previous results. The results for Models 2 and 3 also confirm the expected assumptions, which verified the robustness of the model results.

### 3.3. Willingness to Pay and Trade-Offs Analysis

The willingness to pay for different vaccination service attributes was calculated using the parameter estimation results from the latent class model presented in Table 8, and the results are shown in Table 11. The WTP reveals notable differences between the three latent classes. Specifically, the resolute executors (Class 1) exhibited a significantly higher WTP for weekend vaccination (CNY 74.499), 50% subsidy (CNY 42.362), and 75% subsidy (CNY 37.168) than the mischievous rebels (Class 2) and the incentivized compliers (Class 3). In contrast, the incentivized compliers have a significantly higher WTP for half-hour travelling distance (CNY 80.153), and home vaccination (CNY 119.532) compared to the resolute executors and the mischievous rebels. Moreover, the mischievous rebels demonstrated a slightly higher WTP for online appointment (CNY 26.333) and imported vaccines (CNY 22.764) than the incentivized compliers.

Weighted by the probability of the three classes, the importance of the dog rabies vaccination service attributes varied from high to low: 75% subsidy (CNY 84.709), home vaccination (CNY 53.803), weekend vaccination (CNY 45.480), 50% subsidy (CNY 36.719), half-hour travelling distance (CNY 13.293), online appointment (CNY 7.525), and imported vaccines (CNY 4.165).

**Figure 4:**
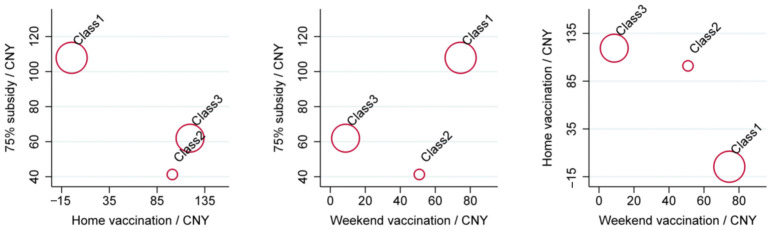
Comparison of the WTP.

By comparing the WTP of the three groups mentioned above, it is evident that the attributes of dog rabies vaccination services have conflicting values in terms of utility for dog-keeping households. Figure 4 illustrates the WTP position of the three latent classes for 75% subsidy, home vaccination, and weekend vaccination, with the size of each circle representing the proportion of each class of dog-keeping households. The proportion of mischievous rebels (Class 2) is significantly smaller than the other two classes, with positive WTP for 75% subsidy, home vaccination, and weekend vaccination. Moreover, the mischievous rebels assign a higher value to weekend vaccination than to the 75% subsidy.

Compared to home vaccination and weekend vaccination, resolute executors (Class 1) placed greater importance on the 75% subsidy attribute, exhibiting a much higher WTP for this attribute than the other two classes. On the other hand, the incentivized compliers (Class 3) have a significantly lower WTP for weekend vaccination at CNY 8.726, but a much higher WTP for home vaccination at CNY 119.532.

## 4. Discussions and Policy Implications

### 4.1. Discussions

Rabies remains a significant public health problem in China and, over the last decade, the country has invested inexhaustive human and financial resources and achieved significant results in rabies prevention and control. This has been made possible by the introduction of large-scale mandatory rabies vaccination for dogs. However, the decline in rabies deaths could be wrongfully labeled as a great step forward, which leads to subsequent relaxation of the controls. Any ensuing lifting of measures and further promises may potentially pose another “peak” or “epidemic wave” of rabies among the population [24]. We should, therefore, be vigilant in this regard to achieve the blueprint for eliminating dog-mediated rabies by 2030.

Effective health education on the risks of rabies is crucial for preventing and controlling the disease. The descriptive statistics presented in this paper indicate that a majority of dog-keeping households feel that their local community or village committee provides average, less, or inadequate information on rabies prevention and control (74.09%). There are various factors that can hinder households from vaccinating their dogs, including being too busy with work, living far away from vaccination points, encountering troublesome vaccination procedures, and facing the high cost of vaccines. It is concerning that 21.06% of households do not comply with the annual vaccination requirements, with urban households accounting for 66.14% of those not vaccinated regularly. The lack of awareness about rabies prevention is a significant challenge in implementing mandatory rabies vaccination policies for dogs. Although a larger sample size is needed to confirm the reliability of our data, our findings are consistent with other studies, such as those by Li et al. [7] and Sambo et al. [25].

Dog vaccination is an effective measure for preventing rabies and can help reduce the costs associated with rabies prevention and control [26]. The current study highlights that enhancing the accessibility of public vaccination services by providing vaccination during non-working hours and in close proximity can increase the marginal utility of rabies vaccination for dog-keeping households. These findings are consistent with previous studies on vaccination programs, such as Mouter et al. [27]. Dog-keeping households are likely to pay a higher premium for more convenient vaccination services, as demonstrated by their WTP an average of CNY 53.803 for home vaccination and CNY 45.480 for weekend vaccination. While there may be a gap between the WTP in the DCE and in reality, the premiums households are willing to pay suggest that there is potential for improving vaccination services.

This study identified significant heterogeneity in the preferences for dog rabies vaccination services, which can be attributed to factors such as residence, having children in households, perception of safety risks, and knowledge of rabies. Although the coefficient for the residence variable in the latent class model was not found to be significant, we believe that urban–rural differences could be an important factor contributing to the preference heterogeneity. The uneven development of public infrastructure in rural and urban areas could explain why most cases of rabies occur in rural areas [28]. Public services in rural areas are underdeveloped, and the return on investment is lower, which limits the ability to carry out tasks such as rescuing stray dogs. Additionally, unlike urban areas where dogs are mainly kept as pets, rural households keep dogs as guards and do not often register or leash them [9,10]. As a result, unregistered and free-roaming dogs in both urban and rural areas are primary hosts of rabies, creating shadow areas for rabies surveillance in China. Addressing these issues will be critical to achieving the goal of eliminating dog-mediated rabies worldwide by 2030.

This study has several limitations that should be acknowledged. First, due to cost and time constraints, the field surveys were only conducted in Guangdong province, which may not be representative of the entire population of dog-keeping households in China. In addition, online surveys may have excluded individuals with lower digital literacy, resulting in sample bias. Second, preferences expressed by respondents in the DCE may not necessarily reflect their actual behavior in real-world scenarios, as they may be influenced by social interactions or other contextual factors. Nonetheless, previous research has shown that DCE is effective in predicting the overall vaccination rate [29], supporting the validity of our findings. Finally, our study was conducted in China, and the results may not be generalizable to other countries with different cultural, social, and economic contexts. Nevertheless, our findings may provide valuable insights for policymakers in emerging countries that are implementing policies to promote dog rabies vaccination and improve rabies prevention and control.

### 4.2. Policy Implications

The empirical findings indicate that there are several implications that could be useful to local governments and decision-makers in enhancing the management of rabies prevention and control by garnering public support.

Improving the accessibility of vaccination services is crucial for effective rabies prevention and control. Firstly, the government should optimize the schedule for vaccination services. For example, they could establish a vaccination appointment system through information networks like WeChat mini-programs and official accounts. In rural areas, they could organize events like a vaccination day to promote the services. Secondly, mobile points for rabies vaccination services should be established for frontline vaccination services. The location of these points should be strategically planned to establish a well-connected service network. Information about these service points should be widely publicized in districts, towns, and villages. The mobile service team could be formed by local institutions for animal epidemic prevention, relevant personnel, rural veterinarians, and animal clinics. They could offer accessible home vaccination services for dog-keeping households. Thirdly, the range of available vaccines could be expanded. One strategy is to use district, town, and village bulletin boards to advertise the manufacturer, production batch, and comprehensive utility of rabies vaccines. We also encourage vaccine competition between domestic and imported manufacturers to optimize the selection mechanism for rabies vaccines.

To sustain the increase in the vaccination rate, it is imperative to enhance dog immunization management. Firstly, the dog registration management system needs to be upgraded to include a comprehensive mapping of dog-keeping households. Dog owners should take responsibility for self-registration and provide information such as the owner’s details, dog information, and vaccination records. The dog ownership registration mechanism should be explored and clarified in accordance with the law. Secondly, effective implementation of rabies vaccination measures can be achieved through public education and awareness-raising campaigns about the risks of rabies. The government should enforce the mandatory dog vaccination program, issue immunization certificates as required, and establish immunization records. Encouraging dog owners to vaccinate their dogs regularly through subsidies and other incentives is also necessary. Thirdly, the authorities should improve their efforts to rescue stray dogs by setting up special funds, improving the social system for capturing, sheltering, and adopting stray dogs, and creating qualified dog shelters and harmless disposal sites.

The third implication is to innovate the approach to dog management. Firstly, it is necessary for the authorities to collaborate with high-tech companies in establishing a dog information management system that includes a database, an APP system, and a unified information platform. This system should utilize electronic identification to manage and share information about dogs and their owners. Intelligent dog tags can also be used for real-time monitoring, identity inquiries, health vaccination, and owner tracking. Secondly, increased monitoring of rabies outbreaks is required. To keep abreast of the rabies epidemic in the region, regular sampling and testing should be conducted using rabies detection kits and antibody detection instruments. The communication between surveillance agencies and primary veterinary stations should be strengthened to promptly report suspected rabies cases. Real-time epidemic monitoring software can be useful in this regard. Thirdly, developing an animal health code system is a valuable option. This system should store electronic immunization information and trace vaccine injections. The health code should be applied to the animal medical system and establish a database of animal electronic medical records.

## 5. Conclusions

Large-scale vaccination is crucial in the fight against rabies outbreaks. In response to the World Health Organization’s call to “eliminate dog-mediated human rabies by 2030”, emerging countries like China must strengthen their political commitment, public education, and strict dog management, while also implementing a viable vaccination program. This paper examines the preferences of 633 dog-keeping households in 21 municipalities in Guangdong province, China, for public services aimed at promoting dog rabies vaccination. The study found that dog-keeping households have significant preferences for accessibility attributes, including vaccination time, location, procedural arrangement, vaccine origin, and government subsidies. These households can be classified into three types: resolute executors (52.13%), mischievous rebels (5.85%), and incentivized compliers (42.02%). The sources of heterogeneity affecting dog-keeping households’ preferences for dog rabies vaccination include the presence of children in the households, the perception of safety risks, and their knowledge of rabies. To improve the convenience and quality of public services, authorities can arrange weekend vaccination, build mobile vaccination stations, and provide home visits for vaccination. Emphasis should also be placed on improving the online appointment system, increasing the vaccine options, and providing diverse subsidies to encourage regular vaccination.

## Figures and Tables

**Table 1:** The attributes and levels used in the discrete choice experiment.

Attribute	Description	Level
Time	Time of rabies vaccination	Vaccination on Monday to Friday
		Weekend vaccination
Place	Location for rabies vaccination	Home vaccination
		Half-hour travelling distance
		One hour or more travelling distance
Appointment	Appointment for rabies vaccination	On-site appointment
		Online appointment
Origin	Origin of the vaccines	Domestic vaccines
		Imported vaccines
Subsidy	Government subsidies for residents to encourage rabies vaccination at vaccine original cost	25% subsidy
		50% subsidy
		75% subsidy
Price	Original price of rabies vaccine (CNY/needle)	25, 50, 75, 100

**Table 2:** Demographic characteristics of the sample.

Demographic Characteristics	Value	Demographic Characteristics	Value
Gender (%)		Annual household income (%)	
Male	36.49	CNY < 10,000	15.17
Female	63.51	CNY 10,000–25,000	14.85
Age (in years) Mean (s.e.)	29.156 (9.184)	CNY 25,000–50,000	11.53
Urban (%)	29.54	CNY 50,000–100,000	13.90
Rural (%)	70.46	CNY 100,000–250,000	27.17
Education (%)		CNY 250,000–500,000	11.69
High school/technical secondary school or below	17.54	CNY > 500,000	5.69
College/higher vocational	15.96	Work related to animals (%)	14.53
Undergraduate	59.72	Living alone (%)	13.59
Postgraduate or above	6.79	Households with children ≤ 12 (%)	45.81

Note: Percentages may total >100% because of rounding.

**Table 3:** Dog ownership and management in the surveyed households.

	Percentage		Percentage
Number of dogs		Management style for domestic dogs	
1	77.57	Tethering	33.18
2	15.96	Cage or captivity	25.59
≥3	6.48	No restrictions on freedom	38.39
Dog ownership time		Other	2.84
1–2 years	25.28		
3–4 years	43.44	Regular annual vaccinations for dogs	79.94
5–6 years	15.01	Factors influencing regular vaccinations for dogs	
≥7 years	16.27	Did not know that vaccination is required	14.38
Domestic dog sources		There will be no problem for dogs kept at home	24.17
From markets, stores or homes that sell them	50.87	Vaccine prices are too expensive	27.80
Rescue station adoptions	4.11	Busy work, no time to vaccinate	42.81
Gifts from friends and family	36.65	Vaccination sites are too far from home	32.86
Picked up	6.16	Troublesome vaccination procedures	29.38
Other	2.21	No suitable tools (e.g., dog crates, etc.) to transport dogs to the vaccination site	20.06
		Other	13.59
Dog risk alert	76.15		
		Dog application	32.70
Reasons for having a dog		Dog registration	46.92
Housekeeping (watch the door)	39.18	Rabies prevention and control publicity	
Spending time with family	71.72	Never	10.11
Market sales	1.42	Less	34.28
Personal preference	63.03	General	29.70
Cultivating love in children	19.75	More	22.12
Other	3.79	Always	3.79

Note: Percentages may total >100% because of rounding.

**Table 4:** Perception of the safety risks related to dogs.

Item	Mean	Standard Deviation	Reliability	Total Reliability
Dog shedding easily causes human allergies.	3.330	1.009	0.686	
People who raise dogs are susceptible to diseases.	2.370	1.078	0.686	
People who eat dog meat are prone to diseases.	2.864	1.214	0.770	
I am worried about being bitten or scratched by a dog.	3.065	1.179	0.643	0.720
I am worried about my dog biting or scratching others.	3.368	1.160	0.654	
I am worried about my dog damaging household items.	3.316	1.165	0.660	
I am worried about the increased expenses caused by my dog’s illness.	3.258	1.105	0.696	

**Table 5:** Level of knowledge about rabies among dog-keeping households.

Items	Correct Responses (%)	“Don’t Know” Responses (%)
(1) Conceptual understanding:		
Rabies is a highly infectious disease caused by the rabies virus.	84.68	15.32
Rabies is a disease that can be transmitted between humans and animals.	87.68	12.32
Rabies is a fatal disease with an almost 100% mortality rate.	72.20	27.80
(2) Hosts and transmission routes:		
Only dogs can carry the rabies virus.	81.99	18.01
Healthy-looking dogs cannot carry the rabies virus.	87.68	12.32
The rabies virus can infect susceptible animals, including humans, through a bite or scratch from an infected animal.	91.15	8.85
(3) Prevention:		
Rabies cannot be prevented.	88.78	11.22
The rabies vaccine can effectively prevent rabies.	90.52	9.48
Dogs should receive a rabies vaccine annually.	89.57	10.43
(4) Treatment:		
If the wound from an animal bite is not bleeding, it does not need to be treated.	89.73	10.27
The wound from an animal bite should be immediately rinsed with clean water or soapy water.	81.52	18.48
Rabies immune globulin should be injected within 24 h of an animal bite.	91.00	9.00

**Table 6:** Estimates on the preferences for rabies vaccination services for dogs.

Variables	Mixed Logit Model		Conditional Logit Model
	Mean	Standard Deviation	Coefficient
Price	−0.011 ***		−0.008 ***
	(0.001)		(0.001)
Weekend vaccination	0.412 ***	0.997 ***	0.303 ***
	(0.064)	(0.089)	(0.036)
Based on 1 h or more travelling distance			
Half-hour travelling distance	0.705 ***	0.396 **	0.497 ***
	(0.082)	(0.172)	(0.053)
Home vaccination	1.327 ***	1.137 ***	0.909 ***
	(0.096)	(0.120)	(0.053)
Online appointment	0.141 ***	0.469 ***	0.093 ***
	(0.054)	(0.120)	(0.036)
Imported vaccines	0.131 **	0.929 ***	0.104 ***
	(0.061)	(0.091)	(0.036)
Based on 25% subsidy			
50% subsidy	0.415 ***	−0.024	0.304 ***
	(0.068)	(0.094)	(0.051)
75% subsidy	0.855 ***	0.431 ***	0.611 ***
	(0.078)	(0.134)	(0.052)
No vaccination	−4.460 ***	3.328 ***	−1.816 ***
	(0.474)	(0.344)	(0.106)
LR chi2	540.03		2463.44
Log likelihood	−2670.7937		−2940.8107
AIC	5375.587		5899.621
Observations	11,394		11,394

Note: *** and ** indicate statistical significance at 1% and 5% levels, respectively. The numbers in brackets are standard errors.

**Table 7:** Latent class model classification.

Classes	LLF	Nparam	BIC	CAIC
2	−2774.379	19	5671.316	5690.316
3	−2665.868	29	5518.799	5547.799

**Table 8:** Results of the latent class model.

Variables	Class 1	Class 2	Class 3
Utility function			
Price	−0.004 ***	−0.017 ***	−0.033 ***
	(0.001)	(0.005)	(0.004)
Weekend vaccination	0.264 ***	0.842 ***	0.284 **
	(0.057)	(0.273)	(0.120)
Based on 1 h or more travelling distance			
Half-hour travelling distance	−0.151	0.509	2.606 ***
	(0.096)	(0.383)	(0.311)
Home vaccination	−0.016	1.670 ***	3.886 ***
	(0.096)	(0.377)	(0.391)
Online appointment	−0.016	0.435 *	0.644 ***
	(0.061)	(0.264)	(0.127)
Imported vaccines	−0.044	0.376	0.718 ***
	(0.057)	(0.257)	(0.143)
Based on 25% subsidy			
50% subsidy	0.150 **	0.615 *	0.964 ***
	(0.074)	(0.320)	(0.160)
75% subsidy	0.383 ***	0.683 **	2.016 ***
	(0.086)	(0.342)	(0.248)
No vaccination	−3.769 ***	2.068 ***	−1.252 ***
	(0.277)	(0.554)	(0.421)
Classification membership function			
Urban	−0.249	−0.335	0.000
	(0.256)	(0.429)	
Child	−0.138	0.886 *	0.000
	(0.227)	(0.454)	
Safety risks perception	0.311	−0.885 **	0.000
	(0.226)	(0.440)	
Knowledge of rabies	−0.863 ***	−1.320 ***	0.000
	(0.247)	(0.413)	
Constant	1.289 **	−1.679	0.000
	(0.625)	(1.123)	
Dog-keeping households	633		
Observations	11,394		
Log likelihood	−2646.0116		
AIC	5366.023		
BIC	5637.634		
Shares (%)	52.13	5.85	42.02

Note: ***, ** and * indicate statistical significance at 1%, 5%, and 10% levels, respectively. The numbers in brackets are standard errors.

**Table 9:** Demographic characteristics for the three types of dog-keeping households.

Demographic Characteristics		Class 1	Class 2	Class 3
		Resolute Executors	Mischievous Rebels	Incentivized Compliers
Residence	Rural (%)	32.73	35.14	24.81
	Urban (%)	67.27	64.86	75.19
Child	No (%)	50.30	24.32	43.23
	Yes (%)	49.70	75.68	56.77
Safety risks perception	Low (%)	39.09	70.27	48.50
	High (%)	60.91	29.73	51.50
Knowledge of rabies	Low (%)	47.27	54.05	22.18
	High (%)	52.73	45.95	77.82

**Table 10:** Results of the robustness tests in the latent class model.

Attribute		Model 1			Model 2			Model 3	
	Class 1	Class 2	Class 3	Class 1	Class 2	Class 3	Class 1	Class 2	Class 3
Utility function									
Price	−0.004 ***	−0.016 ***	−0.033 ***	−0.003 **	−0.017 ***	−0.032 ***	−0.003 **	−0.015 ***	−0.033 ***
	(0.001)	(0.005)	(0.004)	(0.001)	(0.005)	(0.004)	(0.001)	(0.005)	(0.004)
Weekend vaccination	0.265 ***	0.849 ***	0.280 **	0.264 ***	0.856 ***	0.278 **	0.250 ***	0.857 ***	0.319 ***
	(0.057)	(0.276)	(0.121)	(0.058)	(0.276)	(0.116)	(0.058)	(0.285)	(0.120)
Based on 1 h or more travelling distance									
Half-hour travelling distance	−0.147	0.527	2.624 ***	−0.158	0.580	2.532 ***	−0.136	0.425	2.533 ***
	(0.096)	(0.392)	(0.311)	(0.097)	(0.381)	(0.300)	(0.099)	(0.397)	(0.319)
Home vaccination	−0.011	1.690 ***	3.906 ***	−0.028	1.703 ***	3.802 ***	−0.022	1.594 ***	3.854 ***
	(0.096)	(0.388)	(0.390)	(0.097)	(0.380)	(0.381)	(0.101)	(0.388)	(0.406)
Online appointment	−0.014	0.440 *	0.645 ***	−0.026	0.421	0.651 ***	−0.017	0.466 *	0.629 ***
	(0.061)	(0.264)	(0.129)	(0.062)	(0.265)	(0.125)	(0.063)	(0.270)	(0.128)
Imported vaccines	−0.040	0.356	0.713 ***	−0.049	0.394	0.702 ***	−0.044	0.303	0.721 ***
	(0.056)	(0.259)	(0.144)	(0.057)	(0.253)	(0.140)	(0.057)	(0.266)	(0.149)
Based on 25% subsidy									
50% subsidy	0.153 **	0.612 *	0.966 ***	0.144 *	0.651 **	0.953 ***	0.144 *	0.601 *	0.968 ***
	(0.074)	(0.321)	(0.162)	(0.075)	(0.321)	(0.157)	(0.077)	(0.330)	(0.163)
75% subsidy	0.387 ***	0.677 **	2.021 ***	0.372 ***	0.739 **	1.984 ***	0.367 ***	0.663 *	2.012 ***
	(0.086)	(0.345)	(0.250)	(0.087)	(0.351)	(0.243)	(0.091)	(0.351)	(0.257)
No vaccination	−3.763 ***	2.066 ***	−1.257 ***	−3.773 ***	2.091 ***	−1.349 ***	−3.833 ***	2.099 ***	−1.152 ***
	(0.283)	(0.568)	(0.442)	(0.281)	(0.563)	(0.425)	(0.284)	(0.561)	(0.412)
Classification membership function									
Urban	−0.251	−0.376	0.000	−0.284	−0.375	0.000	−0.384	−0.593	0.000
	(0.256)	(0.431)		(0.255)	(0.421)		(0.246)	(0.420)	
Child				−0.199	0.918 **	0.000	−0.227	0.810 *	0.000
				(0.222)	(0.447)		(0.219)	(0.468)	
Safety risks perception	0.336	−0.941 **	0.000				0.248	−1.029 **	0.000
	(0.222)	(0.440)					(0.219)	(0.446)	
Knowledge of rabies	−0.885 ***	−1.260 ***	0.000	−0.849 ***	−1.352 ***	0.000			
	(0.247)	(0.414)		(0.244)	(0.405)				
Constant	1.095 **	−0.133	0.000	1.579 ***	−2.001 *	0.000	1.095 *	−1.942 *	0.000
	(0.502)	(0.758)		(0.598)	(1.108)		(0.597)	(1.140)	
Dog-keeping households		633			633			633	
Observations		11,394			11,394			11,394	
Log likelihood		−2649.1530			−2650.8918			−2654.7783	
AIC		5368.306			5371.784			5379.557	
BIC		5625.235			5628.713			5636.486	

Note: ***, ** and * indicate statistical significance at 1%, 5%, and 10% levels, respectively. The numbers in brackets are standard errors.

**Table 11:** WTP for dog rabies vaccination service attributes.

Attribute	Class 1	Class 2	Class 3	WTP Weighted by Probability
	Resolute Executors	Mischievous Rebels	Incentivized Compliers	
Weekend vaccination	74.499	50.887	8.726	45.480
Half-hour travelling distance	−42.560	30.748	80.153	13.293
Home vaccination	−4.474	100.999	119.532	53.803
Online appointment	−4.478	26.333	19.797	7.525
Imported vaccines	−12.363	22.764	22.081	4.165
50% subsidy	42.362	37.168	29.656	36.719
75% subsidy	107.882	41.297	62.005	84.709

## Data Availability

All study data used for analysis are available upon request.

## References

[1] Knobel D.L., Cleaveland S., Coleman P.G., Fevre E.M., Meltzer M.I., Miranda M.E.G., Shaw A., Zinsstag J., Meslin F.X. (2005). Re-evaluating the burden of rabies in Africa and Asia. Bull. World Health Organ..

[2] Schnell M.J., McGettigan J.P., Wirblich C., Papaneri A. (2010). The cell biology of rabies virus: Using stealth to reach the brain. Nat. Rev. Microbiol..

[3] Warrell M.J. (2016). The dilemma of managing human rabies encephalitis. Trop. Med. Int. Health.

[4] Baxter J.M. (2012). One in a million, or one in thousand: What is the morbidity of rabies in India?. J. Glob. Health.

[5] Rupprecht C.E., Mani R.S., Mshelbwala P.P., Recuenco S.E., Ward M.P. (2022). Rabies in the Tropics. Curr. Trop. Med. Rep..

[6] Yang C., Zhang S., Meng X.Y., Deng X.C., Jiang H., He J.Z., Zhang Y.Y., Wu J., Xie R.Q. (2022). Health risk perception and prevention awareness related rabies among rural middle school in Guangxi Province. Chin. Cent. Health Educ..

[7] Li D.D., Liu Q.Y., Chen F., Jiang Q.Q., Wang T.T., Yin X.X., Lu Z.X., Cao S.Y. (2021). Knowledge, attitudes, and practices regarding rabies and its prevention and control among bite victims by suspected rabid animals in China. One Health.

[8] Miao F.M., Li N., Yang J.J., Chen T., Liu Y., Zhang S.F., Hu R.L. (2021). Neglected challenges in the control of animal rabies in China. One Health.

[9] Tian H.Y., Yun F., Vrancken B., Cazelles B., Tan H., Gill M.S., Yang Q.Q., Li Y.D., Yang W.H., Zhang Y.Z. (2018). Transmission dynamics of re-emerging rabies in domestic dogs of rural China. PLoS Pathog..

[10] Tu C., Feng Y., Wang Y. (2018). Animal rabies in the People’s Republic of China. Rev. Sci. Tech.-Off. Int. Epizoot..

[11] Zhang J.Y., Zhang B., Zhang S.F., Zhang F., Li N., Liu Y., Hu R.L. (2017). Dog-transmitted rabies in Beijing, China. Biomed. Environ. Sci..

[12] Yin W.W., Dong J., Tu C.C., Edwards J., Guo F.S., Zhou H., Yu H.J., Vong S. (2013). Challenges and needs for China to eliminate rabies. Infect. Dis. Poverty.

[13] Ryan M. (2004). Discrete choice experiments in health care. BMJ-Br. Med. J..

[14] McPhedran R., Gold N., Bemand C., Weston D., Rosen R., Scott R., Chadborn T., Amlot R., Mawby M., Toombs B. (2022). Location, location, location: A discrete choice experiment to inform COVID-19 vaccination programme delivery in the UK. BMC Public Health.

[15] Makabayi-Mugabe R., Musaazi J., Zawedde-Muyanja S., Kizito E., Namwanje H., Aleu P., Charlet D., Lopez D.B.F., Brightman H., Turyahabwe S. (2022). Developing a patient-centered community-based model for management of multi-drug resistant tuberculosis in Uganda: A discrete choice experiment. BMC Health Serv. Res..

[16] De Bekker-Grob E.W., Ryan M., Gerard K. (2012). Discrete choice experiments in health economics: A review of the literature. Health Econ..

[17] Lancsar E., Louviere J. (2008). Conducting Discrete Choice Experiments to Inform Healthcare Decision Making. PharmacoEconomics.

[18] Salloum R.G., Shenkman E.A., Louviere J.J., Chambers D.A. (2017). Application of discrete choice experiments to enhance stakeholder engagement as a strategy for advancing implementation: A systematic review. Implement. Sci..

[19] Cummings J.L., Masterman D.L. (1999). Depression in patients with Parkinson’s disease. Int. J. Geriatr. Psychiatry.

[20] Johnson R., Orme B. (2003). Getting the Most from CBC, Sawtooth Software Research Paper Series.

[21] Rose J.M., Bliemer M.C.J. (2013). Sample size requirements for stated choice experiments. Transportation.

[22] Lim K.H., Hu W., Maynard L.J., Goddard E. (2013). US consumers’ preference and willingness to pay for country-of-origin-labeled beef steak and food safety enhancements. Can. J. Agric. Econ. /Rev. Can. D’agroecon..

[23] Mao B., Ao C., Ning J., Gao Q. (2018). A study on the heterogeneity of public ecological preference of wetland ecosystem services based on latent classification model. Nat. Resour. J..

[24] Wu X.F., Hu R.L., Zhang Y.Z., Dong G.M., Rupprecht C.E. (2009). Reemerging Rabies and Lack of Systemic Surveillance in People’s Republic of China. Emerg. Infect. Dis..

[25] Sambo M., Lembo T., Cleaveland S., Ferguson H.M., Sikana L., Simon C., Urassa H., Hampson K. (2014). Knowledge, Attitudes and Practices (KAP) about Rabies Prevention and Control: A Community Survey in Tanzania. PLoS Negl. Trop. Dis..

[26] Xie M., Pan H., Luo Q.J., He D.Z., Su P., Yang Q., Cai H., Huang J.C., Huang Z.L. (2022). The etiology and comprehensive prevention and control of canine rabies. Chin. Livest. Poult. Breed..

[27] Mouter N., Boxebeld S., Kessels R., Wijhe M.V., Wit A.D., Lambooij M., Exel J.V. (2022). Public Preferences for Policies to Promote COVID-19 Vaccination Uptake: A Discrete Choice Experiment in The Netherlands. Value Health.

[28] Qi L., Su K., Shen T., Tang W.G., Xiao B.Z., Long J., Zhao H., Chen X., Xia Y., Xiong Y. (2018). Epidemiological characteristics and post-exposure prophylaxis of human rabies in Chongqing, China, 2007–2016. BMC Infect. Dis..

[29] De Bekker-Grob E.W., Donkers B., Bliemer M.C.J., Veldwijk J., Swait J.D. (2020). Can healthcare choice be predicted using stated preference data?. Soc. Sci. Med..

